# Exosomal circRNAs: Emerging Players in Tumor Metastasis

**DOI:** 10.3389/fcell.2021.786224

**Published:** 2021-12-08

**Authors:** Hao Zhou, Xiaoyun He, Yuxiang He, Chunlin Ou, Pengfei Cao

**Affiliations:** ^1^ Department of Hematology, Xiangya Hospital, Central South University, Changsha, China; ^2^ Departments of Ultrasound Imaging, Xiangya Hospital, Central South University, Changsha, China; ^3^ Department of Oncology, Xiangya Hospital, Central South University, Changsha, China; ^4^ Department of Pathology, Xiangya Hospital, Central South University, Changsha, China; ^5^ National Clinical Research Center for Geriatric Disorders, Xiangya Hospital, Central South University, Changsha, China

**Keywords:** tumor metastasis, exosomes, circRNAs, tumor microenvironment, biomarkers, therapy

## Abstract

Metastasis is an important feature of malignant tumors, and is the primary cause of poor prognosis and treatment failure, in addition to representing a potentially fatal challenge for cancer patients. Exosomes are small extracellular vesicles 30–150 nm in diameter that transmit cargo, such as DNA, RNA, and proteins, as a means of intercellular communication. Exosomes play crucial roles in a range of human diseases, especially malignant tumors. A growing number of studies have verified that circRNAs can be enveloped in exosomes and transferred from secretory cells to recipient cells, thereby regulating tumor progression, especially tumor metastasis. Exosomal circRNAs regulate tumor cell metastasis not only by regulating the signaling pathways, but also by affecting the tumor microenvironment. Moreover, exosomal circRNAs have the potential to serve as valuable diagnostic biomarkers and novel therapeutic targets in cancer patients. In this review, we summarize the mechanism by which exosomal circRNAs modulate metastatic phenomena in various types of tumors, and put forward the prospects of clinical applications of exosomal circRNAs in tumor therapy.

## Introduction

Metastasis is one of the ten essential characteristics of malignant tumors and a hot research topic (Hanahan and Weinberg, 2011). It is a process that enables malignant cells to escape from the primary tumor site, migrate through the lymphatic and/or blood circulation, and ultimately spread to remote sites (Lazebnik, 2010; Tarin, 2011; Sun et al., 2015). The onset of tumor metastases is often indicative of poor prognosis, and >90% of the cancer-related deaths result from metastases (Chambers et al., 2002; Maishi and Hida, 2017; Jiang et al., 2019; Mattiuzzi and Lippi, 2020; Wang et al., 2020d). Most metastatic lesions cannot be surgically eradicated because such lesion is often indicative of more widespread systemic disease (Gupta and Massagué, 2006). Although an increasing number or tumor treatments are being developed with advances in medical technology, tumor metastasis remains one of the major causes of the extremely high mortality rate in a variety cancers. Therefore, the search for tumor markers and therapeutic targets remains an important strategy for improved cancer treatment (Sun L. et al., 2019; Stoletov et al., 2020; Wang J. et al., 2020).

Exosomes are extracellular vesicles that originate from the multivesicular bodies (MVBs) and are present in intercellular space or circulate in biological fluids (Kalluri, 2016; Zhang and Yu, 2019; He et al., 2021a). These vesicles can be internalized by neighboring cells or by remote receptor cells through fusion with the target cell membrane, thereby altering the behavior of the target cell (Hessvik and Llorente, 2018; Gonda et al., 2019). Exosomes play integral roles in mediating intercellular communication, regulating immune system function (Gao et al., 2018; Yu et al., 2018), promoting cell development and differentiation (Zhou et al., 2021), influencing viral replication, and other physiological or pathological disease processes (Alenquer and Amorim, 2015) that affect the progression of many diseases, including tumors (Zhang and Yu, 2019). Recent studies have revealed that exosomes are involved in regulating several malignant biological behaviors of tumors by transporting various growth factors, proteins, lipids, nucleic acids, non-coding RNAs, and other molecules (Kalra et al., 2016), including promoting malignant proliferation, metastasis, and immune escape by tumor cells (Wortzel et al., 2019; Kugeratski and Kalluri, 2021), and contributing to tumor microenvironment (TME) (Meng et al., 2019; Wu et al., 2019). The roles played by non-coding RNAs (ncRNA)—a class of molecules present in high concentrations in exosomes, i.e., microRNAs (miRNAs), long non-coding RNAs (lncRNAs), and circular RNAs (circRNAs)—in tumor metastasis are gradually gaining attention (Zhao et al., 2015; Chen et al., 2020a; Guo et al., 2020). CircRNAs are covalently closed ncRNA molecules, comprising 3′ and 5′ ends joined in a non-collinear manner by reverse splicing (Zhang et al., 2016), which exhibit properties and functions different from those of linear RNA. Many studies have demonstrated that intracellular circRNAs can regulate tumor metastasis in multiple ways. However, the effect of exosomal circRNAs on tumor metastasis cannot be fully explained. Herein, we performed a systematic literature review of exosomal circRNAs in the context of development and progression of tumor metastases.

## Tumor Metastasis

Metastasis of tumor cells is a multi-step process (He et al., 2021b) that includes the following steps: (I)tumor cells lose adhesion to neighboring cells and detach from the primary tumor, a phenomenon that results in the degradation of the extracellular matrix (ECM), and the migration and invasion of the cells into the surrounding tissues (Friedl and Wolf, 2003); (II)infiltration of the tumor cells into the bloodstream, followed by adaptation and escape from anoikis to survive in the circulation (Zhan et al., 2004), (III) exudation of the tumor cells outside the blood vessels (IV), and finally entry of the tumor cells into the metastatic site, followed by adaptation and growth, resulting in the eventual colonization of the site (Fidler, 2003; Nguyen et al., 2009; Scully et al., 2012) (Supplementary Figure S1). Epithelial-to-mesenchymal transition (EMT) is an important form of tumor metastasis. EMT is a complex cellular pathway in which epithelial cells lose intercellular adhesion (characterized by loss of membrane E-Cad) and gain mesenchymal features (characterized by increased N-cadherin expression and migration capacity) (Bakir et al., 2020). Stephen Paget first proposed the classic hypothesis of “seed and soil” in 1989. He compared the primary tumor to a “plant”, the tumor cells to “seeds”, and the host environment to “soil” (Paget, 1989), and boldly hypothesized, “When a plant has seeds, its seeds can be taken anywhere; but they can only survive and grow if they fall on suitable soil”. This assumption is accepted as the fundamental theory for explaining tumors and metastasis (Langley and Fidler, 2011; Ribelles et al., 2014). In this doctrine, it is believed that the autonomous mechanisms of tumor cells are insufficient to accomplish metastasis and that tumor metastasis is regulated by other factors, including tumor microenvironment (TME) (Quail and Joyce, 2013; McAllister and Weinberg, 2014). Interactions between the TME and tumor tissue is gradually gaining attention as a new field (Quail and Joyce, 2013; Liao et al., 2021; Xiao and Yu, 2021). TME refers to the cellular environment in which the tumor exists, and its composition includes tumor cells as well as surrounding blood vessels, ECM, signaling molecules, and non-malignant cells such as fibroblasts and immune cells (Luo et al., 2016). Several studies have shown that the TME has an inhibitory effect on the growth of malignant tumors (Holmgren et al., 1995; Suzuki et al., 2006). However, in most malignant tumors, these inhibitions are overcome, resulting in the use of support cells by malignant tumors to increase their metastatic potential and promote their own growth and relocation to remote sites (Marx, 2013; Massagué and Obenauf, 2016). Tumor cells in highly aggressive primary tumors are more adept at exploiting this particular tissue microenvironment. Moreover, stromal cells and fibroblasts can also secrete growth factors such as hepatocyte growth factor (HGF), chemokines (e.g., CXCL12), and exosomes, which can promote the forming of pre-metastatic niche (PMN) (Filipazzi et al., 2012; Liu and Cao, 2016; Whiteside, 2016). These growth factors not only directly promote the growth and survival of malignant cells, but also act as decoys to stimulate other cells to migrate to the TME and indirectly promote tumor invasion and metastasis (Spaeth et al., 2008; Hanahan and Coussens, 2012). Metastasis is “a long journey” for the tumor cells themselves, as there are many rate-limiting steps in the formation of metastatic cancer, including extravasation, distal organ survival, and the establishment of sustained growth (Psaila and Lyden, 2009; Acharyya and Massague, 2016). Microenvironmental cues play important roles in all steps of metastasis. Thus, successful metastasis depends on the ability of cancer cells to adapt to different microenvironments at each step of the metastatic cascade (primary tumor, body circulation and final metastatic site) (Zhuang et al., 2019).

## Biological Characteristics of Exosomal CIRCRNAS

Exosomes are lipid bilayer nano-vesicles with a “spherical” morphology (30–150 nm) that are thought to be released by almost all cell types (Théry et al., 2002). These vesicles display a number of surface molecular markers, such as CD9, CD63, and CD81 (Mathivanan et al., 2010; Kowal et al., 2016). Exosome formation comprises four stages, i.e., initiation, endocytosis, formation of MVBs, and exosome secretion. In this process, the endosomal sorting complex required for transport (ESCRT) can select ubiquitin-tagged proteins, lead them to MVBs, and separate and release them from the peripheral membrane by a process similar to cytoplasmic division and viral outgrowth (Hanson and Cashikar, 2012; van Niel et al., 2018; Xu et al., 2018). Exosomes are widely available and have become essential mediators of intercellular communication in physiological and pathological states (Meldolesi, 2018; Delpech et al., 2019; Asghar et al., 2020). As a signal vector for intercellular communication, the content of exosomes varies with different physiological and pathological conditions and primary cell types (Bebelman et al., 2018). Many studies have reported that exosomes regulate tumor progression by carrying or delivering multiple biomodulator “cargoes” (including ncRNAs and proteins) (He et al., 2019; Li R. et al., 2019). Exosomes are key contributors to a wide range of biological processes during tumor growth and progression.

Exosomes contain a variety of RNA molecules, including mRNAs, miRNAs, lncRNAs, and circRNAs (Braicu et al., 2015; Yang and Li, 2018). Compared to other types of RNA, circRNAs are abundant and specific non-coding RNAs that are still not fully understood (Nie et al., 2020). CircRNAs was first discovered in Sendai virus and plant-like viruses by electron microscopy in 1976 (Kolakofsky, 1976). It has long been believed that these closed-loop covalent RNA molecules are a byproduct of rare error responses and have no specific functions (Cocquerelle et al., 1993). However, in recent years, many advances have been made in the study of circRNAs: circRNAs are produced by reverse splicing a 3’ splice donor to an upstream 5’ splice acceptor (Wilusz and Sharp, 2013). Because of this specific structure, circRNAs are resistant to exonucleases and exhibit greater stability than linear non-coding RNAs (such as miRNAs and lncRNAs) (Salzman et al., 2012; Jeck et al., 2013). CircRNA can be found in the cytoplasm, nucleus, or extracellular vesicles external to the cell. They usually perform different functions depending on their localization and distribution (Lasda and Parker, 2016; Ou et al., 2020). Although we have not been able to provide a complete explanation for the biological origins and functions of circRNAs, a large number of studies have confirmed that the main function of circRNAs is to effectively sponge miRNAs through a competitive endogenous RNA (ceRNA) mechanism, reduce their inhibitory effect on target genes (Hansen et al., 2013; Hu Y. et al., 2019; Liang et al., 2020), and activate or inhibit downstream signaling pathways by interacting with proteins (Du et al., 2017; Zang et al., 2020). Endogenous circRNAs have been reported to have the potential for translation (Legnini et al., 2017; Lei et al., 2020), and the products of translation may play important roles in disease progression.

CircRNAs can be encapsulated into exosomes that can be shared between cells. The entry of circRNAs into exosomes is influenced in part by the levels of relevant miRNAs in the parent cells (Li et al., 2015). The sorting of circRNAs species into exosomes may be positively regulated. This suggests that circRNAs are selectively encapsulated within exosomes (Hou et al., 2018). Interestingly, related studies have shown that circRNAs are much more enriched in exosomes than in the cells producing them (Dignat-George and Boulanger, 2011; Dou et al., 2016), and that exosomal circRNAs levels are only moderately correlated with the cellular circRNAs levels (Li et al., 2015). Many questions still remain about the mechanisms involved in exosomal circRNAs enrichment. Emerging data indicate that exosomal circRNAs have multiple functions, such as promoting inflammatory responses, regulating hormone levels in the body, and modulating immunity (Table 1). For example, Wang et al. (Wang S. et al., 2020) found that circRNA-0077930 in exosomes released from endothelial cells induces vascular smooth muscle cell senescence in high-glucose environments. This study provides new insights into the mechanism of smooth muscle cell aging in a high-glucose environment. This finding has significant implications for the prevention of diabetic vascular disease and the exploration of new drug targets. Exosomes released from astrocytes deliver circSHOC2 (Chen et al., 2020b) to neuronal cells, thereby ameliorating ischemia-induced apoptosis and protecting neurons from ischemic injury. In case of hypoxia, circHIPK3 (Wang et al., 2020e) is transferred to myocardial endothelial cells *via* exosomes released from cardiomyocytes. CircHIPK3-mediated VEGFA overexpression significantly increases cell proliferation and migration, which preserves the function and integrity of post-infarction myocardial endothelial cells and exhibits cardioprotective effects.

**TABLE 1:** Role of exosomal circRNAs in human diseases.

Diseases	Exosomal circRNA	Secreting cells	Recipient cells	Effect	Ref.
Diabetic retinopathy	CircRNA-cPWWP2A	Vascular epidural cells	Endotropical cells	Involved in diabetes-induced retinal vascular dysfunction	Liu et al. (2019)
Diabetic vascular disease	CircRNA-0077930	Endothelial cells	Vascular smooth muscle cells	Induced vascular smooth muscle cell senescence	Wang et al. (2020c)
Polycystic ovary syndrome	CircLDLR	KGN cells	KGN cells	Regulation of estrogen secretion	Huang et al. (2020b)
Myocardial infarction	CircHIPK3	Cardiomyocytes	Cardiac endothelial cells	Promotes angiogenesis at the border around the infarcted area	Wang et al. (2020e)
Ischemic stroke	CircSHOC2	Astrocyte	Neuronal cells	Inhibits neuronal apoptosis and ameliorates neuronal damage	Chen et al. (2020b)
Intervertebral disc degeneration	CircRNA_0000253	Degenerative nucleus pulposus cells	Normal nucleus pulposus cells	Promotes apoptosis and inhibits proliferation of Normal nucleus pulposus cells	Song et al. (2020)
Osteoarthritis	CircRNA3503	Synovium mesenchymal stem cells	Chondrocyte	Promotes chondrocyte proliferation and migration	Tao et al. (2021)
Breast cancer	Circ_UBE2D2	Triamcinolone-resistant breast cancer cells	Non-triamcinolone-resistant breast cancer cells	Enhance the resistance of breast cancer cells to triamcinolone	Hu et al. (2020)
Glioma	CircRNA-0001445	Glioma cells	Glioma cells	Promotes proliferation and inhibits the apoptosis of glioma cells	Han et al. (2021)

Recently, Li et al. (Li et al., 2015) first revealed the presence and enrichment of circRNAs in cancer-derived exosomes by RNA-seq analysis. CircRNAs play significant roles in regulating tumorigenesis, tumor cell progression, metastasis, and drug resistance development. For instance, Xiao et al. (Dai et al., 2018) transferred circRNA_100284 secreted by arsenite-transformed human liver epithelial cells (L-02) to normal L-02 cells *via* exosomes. CircRNA_100284 acted as a sponge for miRNA-217 to accelerate the cell cycle and promote cell proliferation, thereby inducing malignant transformation of L-02 cells. This mechanism provides a new explanation for arsenite-induced cancer. Glioma-derived exosomal circRNA-0001445 (Han et al., 2021) significantly promotes proliferation and inhibits the apoptosis of glioma cells *via* the miRNA-1275p/SNX5 signaling pathway. Shi et al. (Xiao and Shi, 2020) found that exosomal circRNA_400068 produced by renal cell carcinoma cells promotes the proliferation of healthy kidney cells, significantly inhibits their apoptosis, and promotes their transformation to malignancy, which may be due to signaling through regulation of the miRNA-210-5p/SocS1 axis pathway. Circ_UBE2D2 was found to be upregulated in exosomes isolated from triamcinolone-resistant breast cancer cells (Hu et al., 2020). Exosomes enhance the resistance of breast cancer cells to triamcinolone by mediating the transfer of circRNA_UBE2D2 into non-triamcinolone-resistant breast cancer cells. This mechanism may suggest the prospect of a promising candidate biomarker and therapeutic target for drug resistance in breast cancer.

## Functions and Mechanisms of Exosomal CIRCRNA in Tumor Metastasis

**FIGURE 1:**
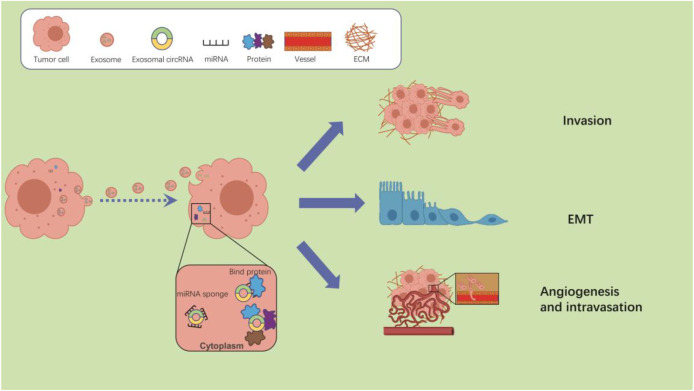
The potential mechanisms of exosomal circRNAs regulate tumor metastasis. After taken up by cancer cells, exosomal circRNAs can regulate the tumor metastasis by interacting with miRNAs or proteins.

Interestingly, researchers have identified that tumor cells produce more than 10 times more exosomes than normal cells. The enrichment and stable presence of circRNAs in exosomes and their intercellular transmission strongly implicate them in tumor metastasis (Li et al., 2015; Bao et al., 2016; Bai et al., 2019). There is increasing evidence that exosomal circRNAs play a key role in the metastasis of various cancers. Exosomal circRNAs may regulate tumor metastasis through a variety of different mechanisms, 1) cytoplasmic circRNAs adsorb miRNAs by sponging and de-repress miRNA-regulated genes, 2) circRNAs delivered to recipient cells can act as a protein sponge or decoy by adsorbing one or more proteins through specific binding sites, 3) exosomal circRNAs are able to regulate the TME through interactions with the immune system cells (Figure 1).

Figure 1 The potential mechanisms of exosomal circRNAs regulate tumor metastasis. After taken up by cancer cells, exosomal circRNAs can regulate the tumor metastasis by interacting with miRNAs or proteins. Exosomal circRNAs regulate tumor metastasis by sponging miRNAs.

CircRNAs represent upstream regulatory molecules with a large number of miRNA response elements (MREs) (Memczak et al., 2013; Lasda and Parker, 2014). CircRNAs can be delivered to recipient cells *via* exosomes and bind to miRNAs, acting as an “miRNA sponge” by blocking the binding of miRNAs to target gene 3’- untranslated regions (UTRs) to disable miRNAs and restore the translation of proteins inhibited by specific miRNA-mRNA binding events, thereby regulating tumor metastasis (Zhong et al., 2018; Verduci et al., 2019). CircRNAs exhibit more preferential binding to miRNAs than other endogenous RNAs (e.g., lncRNAs), hence the name (Shi et al., 2020) “super sponge”. Xie et al. (Xie et al., 2020) found that exosome circSHKBP1 increased HUR expression in gastric cancer (GC) tissues through sponging of miR-582-3p. HUR is reported to be part of the VEGF signaling pathway that promotes VEGF secretion and induces angiogenesis, which promotes GC metastasis. In triple-negative breast cancer (TNBC) (Yang S.-j. et al., 2021, 1), breast cancer cell metastasis is promoted through the direct targeting of AKT1 *via* the exosome circPSMA1 that functions as a sponge for miR-637, which activates the AKT1/*β*-catenin signaling pathway to regulate cell proliferation and migration. High expression of AKT1 and low expression of mir-637 are highly correlated with poor prognosis in TNBC patients with lymph node metastasis. CircPSMA1 overexpression significantly enhances the metastatic capacity in the liver and lungs of mice. Zeng et al. (Zeng et al., 2020) found that overexpression of circFNDC3B severely inhibits angiogenesis in a mouse model of colorectal cancer (CRC). Treatment with exosomal circFNDC3B inhibits CRC cell growth, angiogenesis, and liver metastasis *in vivo*. Liu et al. (Liu et al., 2020) found that circ_MMP2 could be delivered to less invasive hepatocellular carcinoma (HCC) cells *via* exosomes derived from highly invasive HCC cells. Circ_MMP2 can upregulate the expression of its host gene matrix metallopeptidase 2 (*MMP2*) by acting as a sponge for miR-136-5p, a metastasis-associated RNA that promotes HCC cell metastasis.

EMT is one of the initiating steps of primary tumor invasion (Nishiyama et al., 2018; Shang et al., 2019). Exosomal circRNAs can promote tumor metastasis by sponging miRNAs, thereby promoting the EMT phenotype. For example, circPRMT5 (Chen et al., 2018) is expressed at abnormally high levels in urothelial carcinoma of the bladder (UCB) cells, and can be secreted into the blood and urine *via* exosomes. High levels of exosomal circPRMT5 in the serum and urine are positively correlated with lymph node metastasis and tumor progression. CircPRMT5 contributes to a significant reduction in SNAIL1 downregulation by reducing the inhibitory effect of miR-30c, which in turn promotes UCB cell invasiveness and EMT. Statistical analyses showed that high expression of circPRMT5 in UCB tissues is positively correlated with late T and N stages, and is associated with low disease-free survival (DFS). CircNRIP1 is a promoter of EMT in GC (Zhang et al., 2019). The exosomal circNRIP1 sponges miR-149-5p to regulate the expression of AKT1 in GC, which in turn exerts a tumor-promoting effect in the context of EMT. Circ_PVT1 (Wang H. et al., 2020) can enter exosomes originating in cervical cancer cells and function as a tumor promoter by inducing EMT in cervical cancer cells by targeting miR-1286, which in turn promotes tumor metastasis (Table 2).

**TABLE 2:** Regulatory model of “Exosomal circRNA-miRNAs-mRNA” in tumor metastasis.

Tumor types	Exosomal circRNA	miRNA	Targeted gene	Signaling pathways	Ref.
GC	CircSHKBP1	miR-582-3p	HUR	VEGF signaling pathway	Xie et al. (2020)
	CircNRIP1	miR-149-5p	AKT1	mTOR pathway	Zhang et al. (2019)
HCC	Circ-ZNF652	miR-29a-3p	GUCD1	—	(Li et al. (2020b)
	CircPTGR1	miR-449a	MET	—	Wang et al. (2019a)
	Circ_MMP2	miR-136-5p	MMP2	—	Liu et al. (2020)
NSCLC	Circ_100395	miR-141-3p	LATS2	Hippo/YAP signaling pathway	Zhang et al. (2021)
	CircSETDB1	miR-7	sp1	—	Xu et al. (2021a)
	CircARHGAP10	miR-638	FAM83F	—	Jin et al. (2019)
CRC	CircPACRGL	miR-142-3p/miR-506-3p	TGF-*β*1	—	Shang et al. (2020)
	CircFNDC3B	miR-937-5p	TIMP3	VEGF signaling pathway	Zeng et al. (2020)
	CircIFT80	miRNA-1236-3p	HOXB7	—	Feng et al. (2019)
	Circ-133	miR-133a	GEF-H1/RhoA	—	Yang et al. (2020)
Ovarian Cancer	CircRNA051239	miR-509-5p	PRSS3	—	Ma et al. (2021)
Prostate Cancer	Circ_0044516	miR-29a-3p	_	—	Li et al. (2020a)
Triple-negative breast cancer	CircPSMA1	miR-63	AKT1	AKT1/*β*-catenin signaling pathway	Yang et al. (2021b)
Esophageal Cancer	Circ-048117	miR-140	TLR4	—	Lu et al. (2020)
Cervical Cancer	Circ-PVT1	miR-1286	—	—	Wang et al. (2020a)
Pancreatic Cancer	Circ-IARS	miR-122	RhoA	—	Li (2018)
	CircPED8A	miRNA-338	MACC1	MET/AKT and ERK pathway	Li et al. (2018b)
Laryngeal squamous cell carcinoma	CircRASSF2	miRNA-302b-3p	IGF-1R	—	Linli et al. (2019)

Exosomal circRNAs regulate tumor metastasis by binding proteins.

RNA binding proteins (RBPs), a class of proteins involved in gene transcription and translation, are essential elements of circRNA function. Bioinformatic analysis of circRNA sequences reveals a low enrichment of RBP-binding sites compared to the corresponding linear RNAs. However, the unique tertiary structure of circRNA leads to a greater protein binding capacity than linear RNA sequences, allowing better interaction with proteins (Hentze and Preiss, 2013; You et al., 2015; Liang et al., 2018; Huang A. et al., 2020). CircRNAs can function as protein sponges (Yang et al., 2017), decoys (Abdelmohsen et al., 2017), scaffolds, or recruiters (Zeng et al., 2017; Sun Y.-M. et al., 2019) in different physiological and pathological environments. CircRNA-protein interactions also play pivotal roles in the regulation of tumor metastasis.

In HCC, the exosomal circ-0004277 (Zhu et al., 2021) derived from HCC cells blocks the binding of HuR to *ZO-1* mRNA by competitive interactions with HuR protein, which in turn stimulates EMT progression by inhibiting ZO-1. Moreover, exosome-delivered circ-0004277 induces EMT in adjacent normal cells, further promoting the invasion of HCC cells into the surrounding normal tissues. Similarly, Xie et al. (Xie et al., 2020) found that exosomal circSHKBP1 could not only promote GC progression by regulating the miR-582-3p/HUR/VEGF pathway, but also promotes tumor growth and lung metastasis by sequestering HSP90 away from STUB1. Xu et al. (Xu Y. et al., 2021) explored the function of circ-CCAC1 in cholangiocarcinoma (CAA), and found that circ-CCAC1 could translocate into vascular endothelial cells *via* exosomes and bind to EZH2 in the cytoplasm to inhibit the expression of intercellular junctional proteins (ZO-1 and occludin) that control endothelial cell permeability. Decreased expression of intercellular linker proteins disrupts the vascular endothelial barrier and induces angiogenesis, thereby promoting the formation of pre-metastatic ecological niches and providing a supportive microenvironment for the spread of cancer cells.

## Exosomal Circrna Interacts With the Tumor Microenvironment to Regulate Tumor Metastasis

**FIGURE 2:**
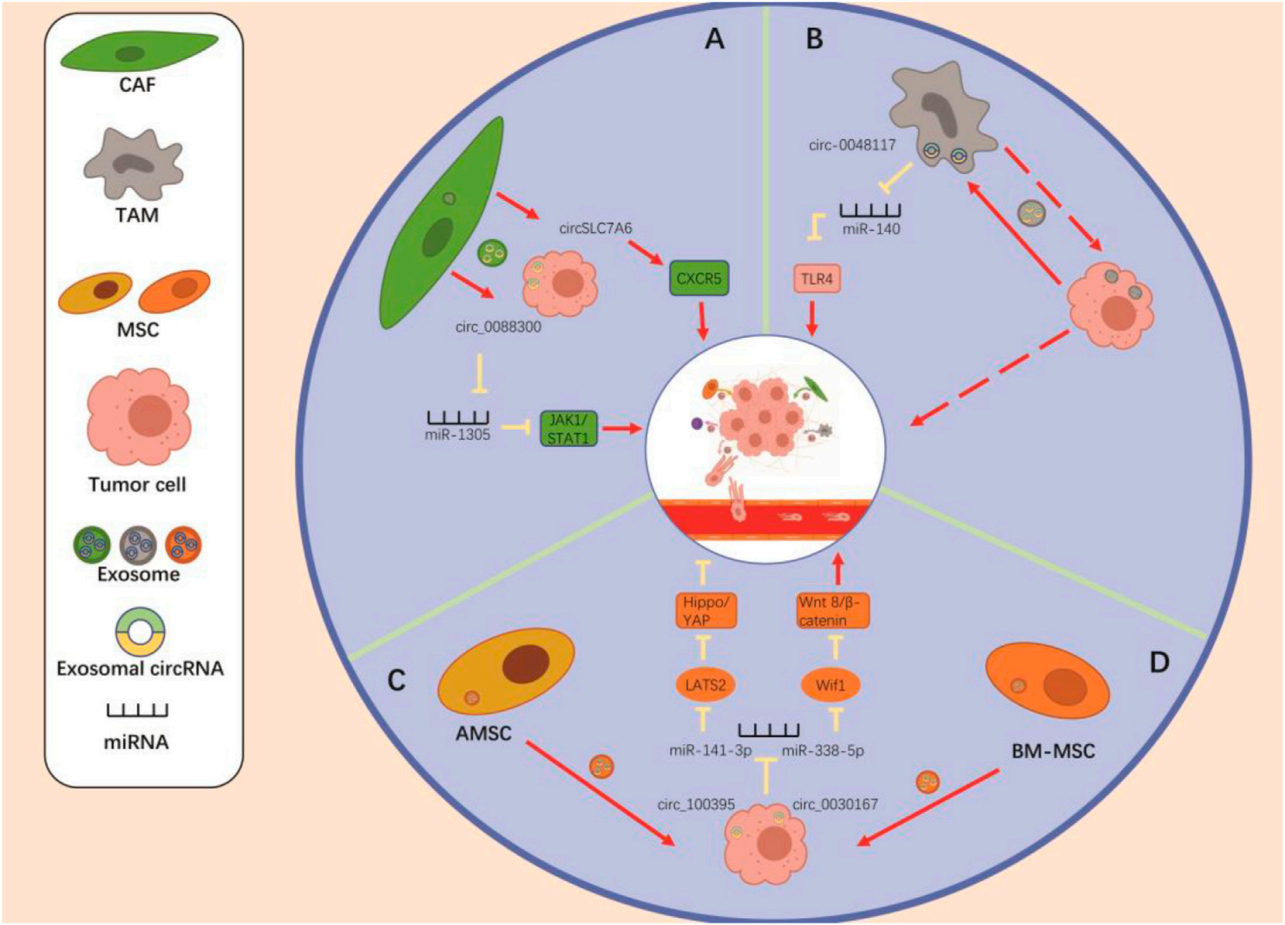
The crosstalk “Non-tumor cells to tumor cells” *via* the exosomal circRNAs to regulate the tumor metastasis. **(A)** CAF-derived exo-circ_0088300 and exo-circSLC7A6 promote the tumor metastasis through regulating the miR-1305/JAK1/STAT1 axis and the expression of CXCR5. **(B)** Tumor-derived exosomal circ-0048117 promotes the polarization of M2 macrophages by regulating the miR-140/TLR4 axis, thereby enhancing the metastatic potential of tumor cells. **(C)** AMSC-derived exosomal circ_100395 inhibits the tumor metastasis *via* regulating the miR-141-3p/LATS2/Hippo/YAP pathway. **(D)** BM-MSC-derived exosomal circ_0030167 promotes tumor metastasis by regulating the miR-338-5p/Wif1/WNT8/*β*-catenin axis in tumor cells. Regulation of mesenchymal stem cells promotes tumor metastasis.

The TME is complicated and ever-evolving. In addition to stromal cells, fibroblasts, and endothelial cells, the TME includes both innate and adaptive immune cells (Hinshaw and Shevde, 2019). Adaptive immune cells are mainly T lymphocytes, which can directly contribute to, or stimulate other cells in the TME to influence tumor growth. They can be classified as “Th1” and “Th2” cells based on their differentiation status. Th1 cells control the pro-inflammatory phenotype, and Th2 cells coordinate the immunosuppressive phenotype (McGuirk and Mills, 2002). The innate immune cell types include macrophages, dendritic cells (DCs), neutrophils, myeloid-derived suppressor cells (MDSCs), natural killer cells (NK), and innate lymphocytes (ILC). The innate immune response can have a significant impact on the TME (Joyce and Pollard, 2009) either directly or indirectly (through control of T-cell fate) (Hinshaw and Shevde, 2019). In addition, immune cells interact with stromal cells, thus influencing tumor development (Tlsty and Coussens, 2006). Here, we focus on mesenchymal stem cells (MSCs), macrophages, and tumor-associated fibroblasts (Figure 2).

### Regulation of MSCs Promotes Tumor Metastasis

MSCs are cells with multiple differentiation capabilities that can differentiate into osteoblasts, chondrocytes, adipocytes, and other cells of mesenchymal origin. It is well known that MSCs are recruited to tumor site thanks to the secretion of soluble factors. Based on this phenomenon, MSCs were explored to determine whether they could regulate nearby cancer cells at the primary site (Ridge et al., 2017). MSCs can produce exosomes, which may deliver signaling molecules by acting as paracrine mediators. Therefore, multiple cellular pathways are controlled to regulate tumor cell proliferation, angiogenesis, and metastasis (Zhao et al., 2020). Currently, whether MSC-derived exosomes promote or suppress tumors remains controversial, depending on the source of exosomes AND the model/tumor cells used as recipient for these exosomes (Vakhshiteh et al., 2019).

In recent years, exosomes released by MSCs have emerged as key regulators of tumor progression in various malignancies. Yao et al. (Yao et al., 2021) found that bone marrow MSC (BM-MSC)-derived exosome circ_0030167 enhances Wif1 expression through regulation of miR-338-5p, which in turn inhibits the Wnt8/*β*-catenin pathway, promoting invasion, migration, and proliferation, as well as tumor stemness in pancreatic cancer cells. Its emergence thus offers a new perspective for the treatment of pancreatic cancer. Interestingly, exosomal circ_100395 from adipose-derived MSC (AMSC) (Zhang et al., 2021) adsorbs miR-141-3p to increase the expression of LATS2, a protein that inhibits tumor cell proliferation, promotes apoptosis, and induces YAP phosphorylation, thereby reducing Hippo pathway activity. The YAP/Hippo pathway promotes EMT and progression of non-small cell lung cancer (NSCLC) cells. Therefore, exosomal circ_100395 inhibits progression and metastasis through the Hippo/YAP signaling pathway that regulates the miR-141-3p/LATS2 axis.

### Regulation of Tumor-Associated Macrophages Promote Tumor Metastasis

Macrophages are among the most plastic, versatile cells of the body. However, these characteristics may be exploited by tumors to trigger different functions at different stages of tumor development (Qian and Pollard, 2010). Macrophages present in tumors are commonly referred to as tumor-associated macrophages (TAMs) and exhibit two distinct polarization phenotypes: Classical activation type (M1) and alternative activation type (M2). M1 macrophages promote the early stages of tumorigenesis by creating an inflammatory microenvironment (Mantovani and Sica, 2010). During tumor progression, TAMs undergo a phenotypic switch to the M2 phenotype. TAM cell populations composed of M2 macrophages play roles in clearing cellular debris, enhancing angiogenesis, and promoting tumor invasion and metastasis (Biswas et al., 2008; Maniecki et al., 2012).

Through both *in vivo* and *in vitro* experiments, Wang et al. (Wang et al., 2021) demonstrated that hsa_circ_00074854 secreted by HCC cells can be delivered to macrophages *via* exosomes, inducing macrophage M2 polarization, thereby promoting migration, invasion, and metastasis of HCC cells. In lung cancer, Katopodi et al. (Katopodi et al., 2021) found that upregulation of exosomal circHIPK3/PTK2 expression promotes the differentiation of monocytes into CD163 + M2 macrophages, which may play an important role in directing lymph node metastasis. In addition, Lu et al. (Lu et al., 2020) found that esophageal squamous cell carcinoma cells in a hypoxic microenvironment produced hsa-circ-0048117-rich exosomes, which promote the polarization of M2 macrophages and enhance the invasiveness and metastatic ability of tumor cells. Other researchers have suggested that macrophages represent a part of the malignant cell population in human tumors. Furthermore, studies have confirmed that TAMs also secrete exosomes, and whether TAMs can influence tumor metastasis through exosomal circRNAs is a direction worthy of future investigations. In conclusion, the roles of macrophages in metastasis require further investigation.

### Regulation of Tumor-Associated Fibroblasts Promotes Tumor Metastasis

Fibroblasts are a major multifunctional cell type in connective tissue that deposit ECM and basement membrane components, regulate related epithelial differentiation events and immune responses, and mediate homeostasis (Li Y.-Y. et al., 2018). Cancer-associated fibroblasts (CAFs) are important components of the TME. CAFs are a major source of secretory growth factors, such as VEGF and pro-inflammatory factors, that are thought to contribute to tumor proliferation, invasion, and metastasis (Kato et al., 2018; Fan et al., 2020).

CAFs have been shown to deliver functional circ_0088300 (Shi et al., 2021) to GC cells *via* exosomes, thereby promoting the proliferation, migration, and invasive capacity of such cells. The inhibition of exosomal circ_0088300 may represent a new therapeutic strategy for GC. In addition, CAF-derived exosomes also promote metastasis and invasion of CRC (Hu J. L. et al., 2019; Gu et al., 2020), breast cancer (Chen et al., 2021) and endometrial cancer cells (Bl et al., 2019), but the roles of exosomal circRNAs remain to be explored.

## Exosomal Circrnas as Novel Biomarkers and Targets of Tumor Metastasis

### Exosomal CircRNA may Serve as Novel Biomarkers of Tumors Metastasis

Exosomes have been found to be widely present in various body fluids, including saliva, plasma, urine, breast milk, amniotic fluid, and bile, which is very convenient for non-invasive testing (Kim et al., 2007). CircRNAs are conserved, stable, and cell- and tissue-specific (Perez de Acha et al., 2020). All of these properties provide strong support for exosomal circRNAs as candidate molecular diagnostic and therapeutic prognostic markers, showing good promise for application as molecular markers in non-invasive tests. Many studies have shown that variable expression of exosomal circRNAs in body fluids is associated with tumor metastasis. Zhang et al. (Zhang et al., 2020) reported that the expression of exosomal circSATB2 is associated with lymphatic metastasis in lung cancer. The expression of circSATB2 was higher in exosomes derived from sera of patients with metastatic lung cancer than in non-metastatic lung cancer, and the receiver operating characteristic (ROC) curve analysis showed its high sensitivity and specificity as a blood test for the diagnosis of lung cancer and lung cancer metastasis. It has also been reported that the expression of exosomal hsa_circRNA_0056616 (He et al., 2020) is significantly lower in patients with lymph node metastasis in lung adenocarcinoma than in patients without lymph node metastasis, and its expression is correlated with tumor-lymph node metastasis (TNM) staging. Plasma exosomal hsa_circRNA_0056616 may be a potential biomarker for predicting lymph node metastasis in lung adenocarcinoma, and its expression level may be a valuable biomarker for the treatment of lymph node metastasis in lung adenocarcinoma. There are many similar studies in other cancer models, and here we list the most representative ones (Table 3). Circ-IARS is abundant in plasma exosomes of patients with metastatic pancreatic cancer (Li et al., 2018). Its high expression is associated with tumor vascular infiltration, liver metastasis, and TNM stage. Similarly, Wu et al. (Li Z. et al., 2018) found that high expression of plasma exosomal circ-PDE8A is associated with duodenal and vascular infiltration, or tumor TNM staging in pancreatic ductal adenocarcinoma, which is correlated with tumor progression and prognosis. Exosomal circ-PDE8A may be a useful marker of pancreatic ductal adenocarcinoma progression. In addition, Li et al. (Li et al., 2020) analyzed the expression of circ_0044516 in patients with high or low levels of metastases in prostate cancer. They found that circ_0044516 levels were higher in blood exosomes of highly metastatic prostate cancer cases than in blood exosomes of low metastatic prostate cancer patients. This finding indicates that exosomal circ_0044516 may be used as a serum marker to measure prostate cancer metastasis, opening a new direction for the treatment of this malignancy. Interestingly, exosomes levels are not only meaningful when measured in blood, but also have value when detected in other body fluids. In urinary exosomes from patients with UCB (Chen et al., 2018), the expression of circPRMT5 is substantially increased compared to that in healthy controls, and its expression level is also associated with lymph node metastasis and tumor progression. Although our research surrounding exosomal circRNAs is still in its infancy, many studies have demonstrated the potential of circRNAs as markers of cancer.

**TABLE 3:** Exosomal circRNA serve as novel biomarkers of tumor metastasis.

Tumor types	Exosomal CircRNAs	Sample types	Expression	Relationship to clinicopathological features	AUC	Ref.
NSCLC	CircSATB2	Serum	Upregulated	Distant metastasis	0.797	Zhang et al. (2020)
	Circ-0056616	Plasma	Downregulated	TNM Stages	0.812	He et al. (2020)
	CicHIPK3/PTK2	Serum	Upregulated	Lymph node metastasis	—	Katopodi et al. (2021)
	CircRNA-102481	Serum	Upregulated	TNM Stages; Brain metastasis	—	Yang et al. (2021a)
SCLC	Exo-FECR1	Serum	Upregulated	Lymph node metastasis; Stages	—	Li et al. (2019a)
Nasopharyngeal Carcinoma	CircMYC	Serum	Upregulated	TNM Stages	—	Luo et al. (2020b)
Oral squamous cell carcinoma	Circ_0000199	Serum	Upregulated	TNM Stages	—	Luo et al. (2020a)
Prostate Cancer	Circ_0044516	Serum	Upregulated	—	—	Li et al. (2020)
Pancreatic Cancer	Circ-PDE8A	Plasma	Upregulated	Duodenal infiltration; Vascular invasion; TNM Stages	—	Li et al. (2018b)
	Circ-IARS		Upregulated	Liver metastasis; TNM Stages	—	Li, (2018)
Esophageal Cancer	Circ_0026611	Serum	Upregulated	Lymph node metastasis	0.724	Liu et al. (2021)
	Circ-0048117		Upregulated	TNM Stages	—	Lu et al. (2020)
HCC	Circ-100338	Serum	Upregulated	TNM Stages; Vascular invasion; Pulmonary metastasis	—	Huang et al. (2020c)
UCB	CircPRMT5	Serum/Urine	Upregulated	Lymph node metastasis	—	Chen et al. (2018)
CRC	Circ-0004771	Serum	Upregulated	TNM Stages	0.88	Pan et al. (2019)
Cholangiocarcinoma	Circ_0000284	Plasma	Upregulated	TNM Stages	—	Wang et al. (2019b)
	Circ-CCAC1	Serum	Upregulated	TNM Stages	0.759	Xu et al. (2021b)
		Bile			0.857	

Exosomal circRNAs as targets of tumors cell metastasis.

### Exosomal CircRNAs as Targets of Tumors Metastasis

**FIGURE 3:**
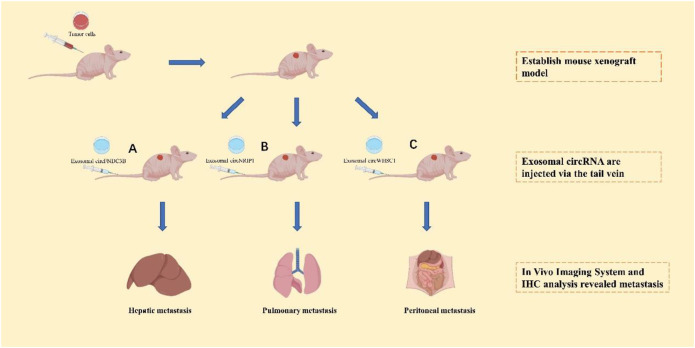
The application of exosomal circRNAs as targets of tumors cell metastasis *in vivo*. After establishing the mouse xenograft model, exosomal circFNDC3B **(A)**, exosomal circNRIP1 **(B)**, and exosomal circWHSC1 **(C)** will be injected into the mouse xenograft model to analyze the organ-spectific metastasis.

Many tumor metastasis-associated exosomal circRNAs have potential clinical applications (Figure 3). By comparing mice injected with circFNDC3B-containing exosomes and normal control exosomes, Zeng et al. (Zeng et al., 2020) found that mice separately injected with exosomal circFNDC3B had reduced tumor volume and weight, and a substantial reduction in VEGFR expression relative to mice injected with normal control exosomes. This result demonstrates that treatment with exosomal circFNDC3B inhibits CRC tumor growth, angiogenesis, and liver metastasis. Zhang et al. (Zhang et al., 2019) examined the role of exosomal circNRIP1 in distant metastasis through tail vein injection of GC cells co-cultured with circNRIP1-containing exosomes and normal control exosomes into BALB/c nude mice. They found that lung and peritoneal metastases were more frequent in nude mice injected with circNRIP1-containing exosomes than in mice treated with normal control exosomes, indicating that exosomal circNRIP1-treated GC cells exhibit greater metastatic potential. To investigate the role of exosomal circWHSC1 in the peritoneal dissemination of ovarian cancer, *Zong et al.* (Zong et al., 2019) injected CAOV3 cells intraperitoneally into nude mice to generate tumors, and then injected exosomes containing circWHSC1 or PBS every 2 days. They found that for the exosome-treated group, the number of peritoneal tumor nodules increased significantly and induced changes in their EMT. Recently, exosome-based transport systems have become an innovative platform for the transport of RNAs (siRNAs, microRNAs) or active-drug substances with enhanced specificity, and greater safety and stability compared to other carriers. Exosomes can be used as novel nanomaterials to deliver cargoes of circRNA inhibitors and agonists for suppressing tumor metastasis. However, we are still in the mapping stage of the clinical application of exosomal circRNAs, and no circRNA drug has entered clinical trials at the time of writing this review. MiRNAs are the most comprehensively understood non-coding RNAs, and several clinical studies have been conducted on miRNA-based interventions with respect to the progression of malignant tumors (Hong et al., 2020). A few examples are the first phase I clinical trial of cobomarsen (anti-miR-155 oligonucleotide) in patients with cutaneous T-cell lymphoma (CTCL) (Seto et al., 2018) and a phase I clinical trial of a miR-16 analogue in patients with malignant pleural mesothelioma (van Zandwijk et al., 2017) or NSCLC. It is believed that, in the near future, circRNAs will also be used in clinical applications.

## Future Perspectives

With the rapid development of high-throughput sequencing technology, an increasing number of exosomal circRNAs have been discovered and identified, and have been studied and proven to play important roles in tumorigenesis. The covalently closed loop structure of circRNAs prevents them from being easily degraded by nucleases. Compared with exosomal proteins and ncRNAs (miRNA, lncRNA), exosomal circRNAs from tissues and blood are more conserved, stable, and exhibit greater target specificity, which is characteristic of tumor metastasis-related markers. In addition, as exosomal circRNAs play important regulatory roles in tumor metastasis, they have significant potential as important candidate targets for tumor metastasis-related therapies, which opens new avenues for curing tumorigenic diseases. As the study of exosomal circRNAs is still in its infancy, many aspects remain limited. 1) The technology for the isolation and purification of exosomes is not yet sufficiently developed. Currently, there are four main methods for isolating and purifying extracellular vesicles, i.e., ultra-high speed centrifugation, ultrafiltration, precipitation, and immune-enrichment (van Niel et al., 2018). It is difficult to distinguish between exosomal and non-vesicular components using established purification methods, and this may affect subsequent experimental procedures involving exosomal circRNA, both *in vivo* and *in vitro*; 2) Although high-throughput sequencing techniques have identified many exosomal circRNAs that are aberrantly expressed in tumor tissues, their specific mechanisms of action and biological functions are still not fully understood; 3) The factors that determine the endogenous and exogenous nature of exosomes remain undetermined, which in part leads to the difficulty in using exosomal circRNAs as clinical markers of tumor metastasis; 4) Although an increasing number of studies have focused on exploring the use of exosomal circRNAs as biological markers for the diagnosis of certain tumors, only a few clinical trials have confirmed their feasibility.

In conclusion, this review has discussed the regulation of the occurrence and transport of exosomal circRNAs in various biological, physiological, or pathological processes by describing them as stellar molecules that have attracted much attention over recent years. Exosomal circRNAs are enriched in tumors and regulate tumor metastasis through mechanisms such as by acting as sponges for miRNAs, binding to proteins, and interacting with the TME. Although our current understanding of the functions of exosomal circRNAs is undoubtedly only the tip of the iceberg, the development of new technologies and assays would enable us to understand the regulatory mechanisms involving exosomal circRNAs, which will provide superior evidence for their use as early, novel markers of tumor metastasis, and as promising candidate therapeutic targets.

## Glossary

AMSC: adipose-derived MSC
CAA: cholangiocarcinoma
CAFs: Cancer-associated fibroblasts
ceRNA: competitive endogenous RNA
circRNAs: circular RNAs
CRC: colorectal cancer
DCs: dendritic cells
DFS: disease-free survival
ECM: extracellular matrix
BM-MSC: bone marrow MSC
EMT: Epithelial-to-mesenchymal transition
ESCRT: endosomal sorting complex required for transport
GC: gastric cancer
HCC: hepatocellular carcinoma
ILC: innate lymphocytes
lncRNAs: long non-coding RNAs
MDSCs: myeloid-derived suppressor cells
miRNAs: microRNAs
MMP2: matrix metallopeptidase 2
MREs: miRNA response elements
MSCs: Mesenchymal stem cells
MVBs: multivesicular bodies
NK cells: natural killer cells
NSCLC: non-small cell lung cancer
PMN: pre-metastatic niche
RBPs: RNA binding proteins
ROC: receiver operating characteristic
SCLC: Small cell lung cancer
TAMs: tumor-associated macrophages
TME: tumor microenvironment
TNBC: triple-negative breast cancer
TNM: tumor-lymph node metastasis
UCB: urothelial carcinoma of the bladder
UTRs: untranslated regions
