# Supplementary material for: Exosomal circRNAs: Emerging Players in Tumor Metastasis
Source: Front Cell Dev Biol. 2021 Dec 8;9:786224. doi: 10.3389/fcell.2021.786224 (PMC8692866; doi:10.3389/fcell.2021.786224)
Supplement: Supplementary file 1 [file DataSheet1.PDF]

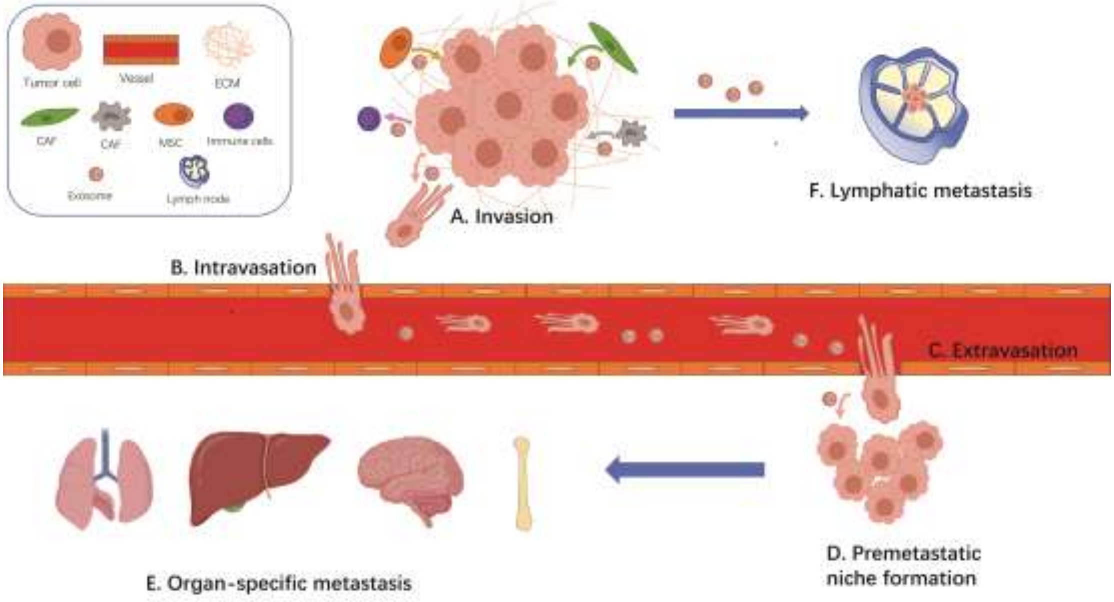

Supplementary Figure 1 The process of tumor metastasis. The process of tumor metastasis contains invasion (**A**), intravasation (**B**), extravasation (**C**), Premetastatic niche formation (**D**), Organ-spectific metastasis (**E**), and lymphatic metastasis (**F**).
